# Supplementary figures and images for: Obesity defined by body mass index and waist circumference and risk of total knee arthroplasty for osteoarthritis: A prospective cohort study
Source: PLoS One. 2021 Jan 7;16(1):e0245002. doi: 10.1371/journal.pone.0245002 (PMC7790262; doi:10.1371/journal.pone.0245002)

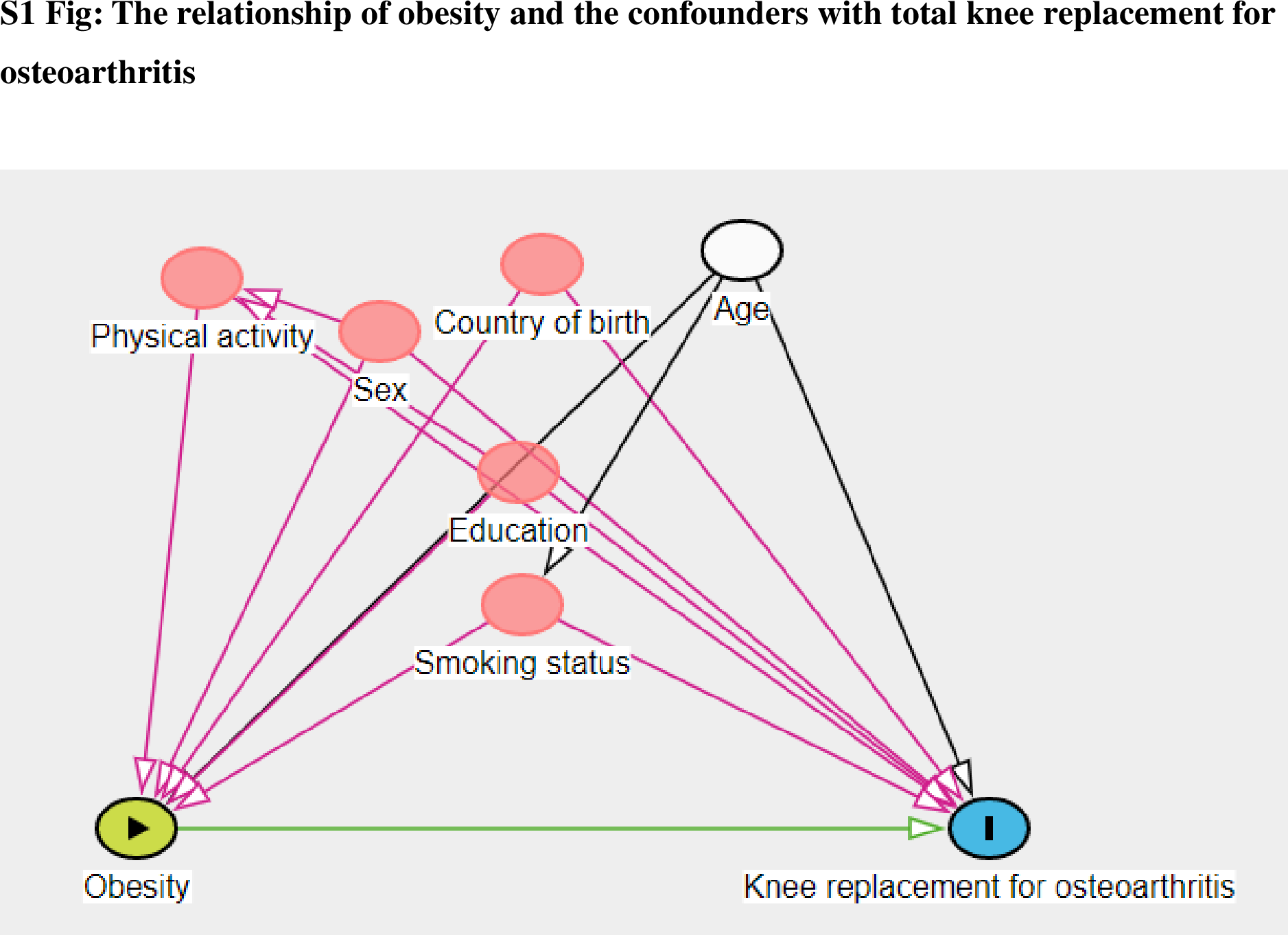

Supplement: S1 Fig — (TIF) [file pone.0245002.s001.tif]

**S1 Appendix: STATA formula for ‘punafcc’**


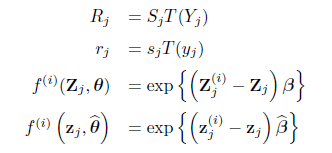

Supplement: S1 Appendix — (DOCX) [file pone.0245002.s004.docx]
