# Supplementary material for: Obesity defined by body mass index and waist circumference and risk of total knee arthroplasty for osteoarthritis: A prospective cohort study
Source: PLoS One. 2021 Jan 7;16(1):e0245002. doi: 10.1371/journal.pone.0245002 (PMC7790262; doi:10.1371/journal.pone.0245002)
Supplement: S1 Table — (DOCX) [file pone.0245002.s002.docx]

**S1_ table. Relationship of different definitions of obesity, and obesity status with incidence of total knee arthroplasty for osteoarthritis**

|  | Model 1  HR (95% CI) | Model 2  HR (95% CI) |
| --- | --- | --- |
| **Men** |  |  |
| Obesity based on BMI | 2.01 (1.74, 2.32) | 2.35 (2.04, 2.72) |
| Obesity based on WC | 2.02 (1.74, 2.33) | 2.20 (1.90, 2.54) |
| Obesity based on Either BMI or WC | 2.02 (1.76, 2.31) | 2.32 (2.02, 2.67) |
| **Obesity status** |  |  |
| No obesity either BMI obesity or WC obesity | 1.00 | 1.00 |
| Obesity is not identified if one of BMI or WC is used | 1.72 (1.44, 2.07) | 2.01 (1.67, 2.41) |
| Obesity based on BOTH BMI and WC | 2.31 (1.96, 2.72) | 2.64 (2.23, 3.11) |
|  |  |  |
| **Women** |  |  |
| Obesity based on BMI | 2.48 (2.26, 2.73) | 2.85 (2.58, 3.15) |
| Obesity based on WC | 2.07 (1.88, 2.29) | 2.30 (2.08, 2.54) |
| Obesity based on Either BMI or WC | 2.31 (2.11, 2.54) | 2.62 (2.38, 2.89) |
| **Obesity status** |  |  |
| No obesity either BMI obesity or WC obesity | 1.00 | 1.00 |
| Obesity is not identified if one of BMI or WC is used | 1.90 (1.66, 2.17) | 2.10 (1.84, 2.40) |
| Obesity based on BOTH BMI and WC | 2.61 (2.35, 2.91) | 3.05 (2.73, 3.41) |

CI, confidence interval; BMI, body mass index; WC, waist circumference

Model 1. adjusted for age

Model 2: adjusted for age, smoking status, physical activity and country of birth
