# Supplementary material for: Obesity defined by body mass index and waist circumference and risk of total knee arthroplasty for osteoarthritis: A prospective cohort study
Source: PLoS One. 2021 Jan 7;16(1):e0245002. doi: 10.1371/journal.pone.0245002 (PMC7790262; doi:10.1371/journal.pone.0245002)
Supplement: S2 Table — (DOCX) [file pone.0245002.s003.docx]

**S2_ table. Estimated population attributable fraction (PAF, %) of total knee arthroplasty in relation to different definitions of obesity**

|  | Model 1 PAF (95% CI) | Model 2 PAF (95% CI) |
| --- | --- | --- |
| Men |  |  |
| Obesity based on BMI | 15.8 (11.0, 18.4) | 17.0 (13.2, 20.6) |
| Obesity based on WC | 13.5 (9.7, 17.0) | 14.7 (11.0, 18.2) |
| Obesity based on either BMI and WC | 18.1 (13.8, 22.2) | 20.5 (16.3, 24.5) |
|  |  |  |
| Women |  |  |
| Obesity based on BMI | 23.9 (20.1, 26.7) | 25.7 (22.8, 28.5) |
| Obesity based on WC | 19.2 (16.2, 22.2) | 20.9 (17.9, 23.8) |
| Obesity based on either BMI and WC | 26.6 (23.2, 29.8) | 28.9 (25.6, 32.0) |

PAF, population attributable fraction; CI, confidence interval; BMI, body mass index; WC, waist circumference

Model 1. adjusted for age

Model 2: adjusted for age, smoking status, physical activity and country of birth
