# Supplementary material for: Obesity defined by body mass index and waist circumference and risk of total knee arthroplasty for osteoarthritis: A prospective cohort study
Source: PLoS One. 2021 Jan 7;16(1):e0245002. doi: 10.1371/journal.pone.0245002 (PMC7790262; doi:10.1371/journal.pone.0245002)
Supplement: Attachment — Submitted filename: Response to Reviewers.docx [file pone.0245002.s006.docx]

**PONE-D-20-22684R1**

**Obesity defined by body mass index and waist circumference and risk of total knee arthroplasty for osteoarthritis: a prospective cohort study**

**Thank you for submitting your manuscript to PLOS ONE. After careful consideration, we feel that it has merit but does not fully meet PLOS ONE’s publication criteria as it currently stands. Therefore, we invite you to submit a revised version of the manuscript that addresses the points raised during the review process.**

**Response:** Thank you for giving the opportunity to revise. We are submitting the revised version.

**Please include the following items when submitting your revised manuscript:**

- **A rebuttal letter that responds to each point raised by the academic editor and reviewer(s). You should upload this letter as a separate file labeled 'Response to Reviewers'.**
- **A marked-up copy of your manuscript that highlights changes made to the original version. You should upload this as a separate file labeled 'Revised Manuscript with Track Changes'.**
- **An unmarked version of your revised paper without tracked changes. You should upload this as a separate file labeled 'Manuscript'.**

**Response:**

- We have uploaded a rebuttal letter. The file is labelled as “response to reviewers”
- We have uploaded a marked copy of the revised manuscript labelled as 'Revised Manuscript with Track Changes'.
- We have also submitted a unmarked version of the manuscript labelled as ‘manuscript’

**If applicable, we recommend that you deposit your laboratory protocols in protocols.io to enhance the reproducibility of your results. Protocols.io assigns your protocol its own identifier (DOI) so that it can be cited independently in the future. For instructions see:** [**http://journals.plos.org/plosone/s/submission-guidelines#loc-laboratory-protocols**](http://journals.plos.org/plosone/s/submission-guidelines#loc-laboratory-protocols)

**Response:** Not applicable

**If you would like to make changes to your financial disclosure, please include your updated statement in your cover letter. Guidelines for resubmitting your figure files are available below the reviewer comments at the end of this letter.**

**Response:** We want to report our financial disclosure as was reported in the cover letter when we submitted the manuscript for the first time. We have uploaded our figure 1 file in the PACE. PACE software reported “DOCX file is converted to a valid TIF file”. We are replacing the previous file with this current TIF file after downloading from PACE. Please let us know if we need to perform further modification.

**Reviewer #4: Dear authors
Congratulations for your done valuable work. I have a short comment, it's better to add the range of age, BMI and waist circumference to table (1) as separate raws with each one under the related variable or between brackets under the related means and SD but not in a separate table. The normal distributed variables are presented as mean, SD and range (minimum and maximum), while the non parametric data are presented as median and interquartile range.

Dood luck**

**Response:** As the reviewer suggested, we have added the range of age, BMI and waist circumference to table (1) between brackets under the related means.
