# Supplementary material for: Obesity defined by body mass index and waist circumference and risk of total knee arthroplasty for osteoarthritis: A prospective cohort study
Source: PLoS One. 2021 Jan 7;16(1):e0245002. doi: 10.1371/journal.pone.0245002 (PMC7790262; doi:10.1371/journal.pone.0245002)
Supplement: Attachment — Submitted filename: Response to reviewer comments.docx [file pone.0245002.s005.docx]

**PONE-D-20-22684**

**Obesity defined by body mass index and waist circumference and risk of total knee arthroplasty for osteoarthritis: a prospective cohort study**

Review Comments to the Author

**Reviewer #1: Congratulations for the great work. Having access to this information bank of your country is a privilege that should be used as you did. Obesity has to be combated by all health professionals and cannot go unnoticed. Making this risk clear is really our role.**

Response: We would like to thank the reviewer for these encouraging comments.

**Reviewer #2: Using a longitudinal study, the authors demonstrated that obesity is a risk factor for total knee arthroplasty for osteoarthritis. They also estimated PAR for obesity based on its different definition. Here are my concerns**.

Response: We would like to respond in line with the reviewer’s comments.

**Method
• The authors stated that they employed a standard questionnaire and cited the reference number 9. Please provide the reliability and validity indices of the study questionnaire.**

Response: Data on date of birth, sex, country of birth, and education were collected based on standard questionnaires that were piloted prior to data collection(1). Smoking was assessed by asking participants if they currently smoke. Participants who reported currently smoking at least seven cigarettes weekly were categorised as current smokers. Those not currently smoking but who had smoked at least seven cigarettes weekly for at least a year were categorised as ex‐smokers. Others were classified as never smokers(2, 3). Physical activity was assessed by three separate questions obtained from the Risk Factor Prevalence Study conducted by the National Heart Foundation and Australian Institute of Health regarding frequency of non-occupational vigorous and moderate physical activity, and walking(2, 4). Participants were categorised as whether or not participating in vigorous physical activity in line with values published in the Compendium of Physical Activities(5). All these suggest the reliability and validity of the study questionnaire.

Author action: We have now clarified the issue raised by the reviewer and amended the text in the manuscript. (page 5, paragraph 1)

*At baseline, demographic and lifestyle data, including date of birth, sex, and country of birth, were collected using pre-piloted standard questionnaires (11). Smoking was assessed by asking participants if they smoked, if so whether they have smoked at least seven cigarettes a week, and were classified as non-smoker or current/ex-smoker(12, 13). Physical activity was assessed by three separate questions obtained from the Risk Factor Prevalence Study conducted by the National Heart Foundation and Australian Institute of Health regarding frequency of non-occupational vigorous and moderate physical activity, and walking(12, 14). Participants were categorised as whether or not participating in vigorous activity in line with values published in the Compendium of Physical Activities(15).*

• **The classification of obesity based on WC and BMI is not clear. Please using a 2 by 2 table, clarify the status of the participants based on these 2 index**.

Response: As we have indicated in our manuscript, we firstly defined obesity based on BMI (BMI ≥30 kg/m^2^) and WC (WC ≥102 cm for men and ≥88 cm for women) separately. Based on these two variables obesity status was classified as ‘no obesity’ (not having obesity based on either BMI or WC); ‘obesity based on both BMI and WC’; and ‘misclassified obesity’ (obesity based on either BMI or WC, but not both). As the reviewer requested, we present the 2 by 2 table below.

|  | Obesity based on WC  **No** | Obesity based on WC  **Yes** | Total |
| --- | --- | --- | --- |
| Obesity based on BMI  **No** | 27056 | 2227 | 29283 |
| Obesity based on BMI  **Yes** | 2063 | 5438 | 7501 |
| Total | 29119 | 7665 | 36784 |

There were 27,056 participants who did not have obesity defined by either BMI or WC, and 5438 participants were defined as obesity based on both BMI and WC.

Based on BMI only, 7501 participants had obesity. However, 2227 participants who were classified as non-obese based on BMI were obese based on WC.

Based on WC only, 7665 participants had obesity. However, 2063 participants who were classified as non-obese based on WC were obese based on BMI.

So 4290 participants were misclassified if only BMI or WC was used to define obesity.

Author action: We have presented these numbers and categories in our manuscript as Figure 1.

**Fig 1: Obesity defined by body mass index and/or waist circumference and discordance between these measures**

2227

(6.1%)

Only WC obesity

2063

(5.6%)

Only BMI obesity

5438

(14.8%)

Both BMI and WC obesity

27,056 (73.6%) No obesity based on both BMI and WC

**• Please provide the confounding criteria utilized for study confounder selection?**

Response: The figure below explains the relationship between obesity and total knee replacement for osteoarthritis with all variables used to build the models. We have selected the model based on the DAG diagram(6).

Author action: We have added the following text (page 6, paragraph 1) and have included the figure as Supplementary Figure in the manuscript.

*Confounders were selected based on the Directed acyclic graph (DAG) diagram(6) (S1 Fig)*

**S1 Fig: The relationship of obesity and the confounders with total knee replacement for osteoarthritis**

For our model building, we first considered the model which included all the predictors that had a p-value of less than 0.2 – 0.25 in the univariate analyses which in this particular analysis meant that we included every predictor in our model (https://stats.idre.ucla.edu/stata/seminars/stata-survival/#building).

Table: the unadjusted association of the following variables with knee replacement

| Explanatory variable | HR (95% CI) | P value |
| --- | --- | --- |
| Sex | 1.26 (1.16, 1.37) | <0.001 |
| Smoking status | 0.89 (0.82, 0.96) | 0.003 |
| Vigorous physical activity | 1.09 (1.01, 1.18) | 0.02 |
| Country of birth | 0.60 (0.54, 0.67) | <0.001 |
| Education | 1.01 (0.94, 1.10) | 0.75 |

All the variables except for education fulfilled these criteria for inclusion in the final regression model. We repeated our analysis without adjustment for education, the results were very similar.

Author action: We have removed education from the list of confounders. Now all our analyses are adjusted for age, sex, smoking status, vigorous physical activity and country of birth.

**• Please add the utilized PARF formula**.

Response: We used the “punafcc” command from STATA that estimates a marginal mean between-scenario risk or hazard ratio for survival data. The formula for PAF is:

∑pd_i_[(HR_i_ − 1)/HR_i_], where pd_i_ is the proportion of total knee replacement for OA observed in the i^th^ obesity category and HR_i_ is the hazard ratio (HR) associated with that category.

In STATA the formula looks like the following

This implies that is the population mean risk ratio (or hazard ratio) between scenario i and the real world for the “subsubpopulation” of cases (or failures) of the subpopulation specified by the subpop() option and that (4) is a corresponding sample mean risk ratio (or hazard ratio) for the “subsubsample” of cases (or failures) of the subsample specified by the subpop() option. A mean between-scenario ratio is a subtly different quantity from a ratio between scenario means; however, both of these quantities are known as population unattributable fractions and can be subtracted from 1 to give population attributable fractions.

Author action: We have added the following line in the statistical analysis section (page 6, paragraph 1). We have also presented the STATA formula as an appendix.

*The formula for PAF used is:*

*∑pd_i_[(HR_i_ − 1)/HR_i_], where pd_i_ is the proportion of total knee replacement for OA observed in the i^th^ obesity category and HR_i_ is the hazard ratio (HR) associated with that category. The STATA formula for ‘punafcc’ is presented as an appendix (S1 Appendix).*

**Results
• Table 1: please add the utilized test for comparison as well as their corresponding Pvalue.**

Response: According to current STROBE guideline for reporting cohort studies, “P values for the table that describes the cohort profile are not essential”(7, 8). In fact in the study published in the PLoS Medicine and the other studies regarding reporting STROBE checklist published in the Lancet, Epidemiology, Annals of Internal Medicine, and the BMJ, a P value was not presented for the descriptive tables they have provided as an example. However, as suggested by the reviewer we have included p values in table 1.

**Table 1. Baseline characteristics of study participants**

|  | **Total population**  **(n=36,784)** | **No knee arthroplasty (n=34,101)** | **Knee arthroplasty**  **(n=2,683)** | **P value**  **No knee arthroplasty vs Knee arthroplasty** |
| --- | --- | --- | --- | --- |
| **Age, years** | 62.6 (8.9) | 62.5 (8.9) | 63.7 (7.8) | <0.001 |
| **Male, n (%)** | 14,888 (40.5) | 13,976 (41.0) | 912 (34.0) | <0.001 |
| **Country of birth, n (%)** |  |  |  | <0.001 |
| **Australia and UK** | 27,705 (75.3) | 25,493 (74.8) | 2,212 (82.4) |  |
| **Italy and Greek** | 9,079 (24.7) | 8,608 (25.2) | 471 (17.6) |  |
| **Secondary education, degree/diploma, n (%)** | 15,606 (42.7) | 14,503 (42.8) | 1,103 (41.3) | 0.12 |
| **Moderate and high level of physical activity, n (%)** | 21,168 (57.6) | 19,569 (57.4) | 1,599 (59.6) | 0.03 |
| **Current/ex-smoker, n (%)** | 15,402 (41.9) | 14,394 (42.2) | 1,008 (37.6) | <0.001 |
| **BMI, kg/m^2^** | 26.9 (4.4) | 26.7 (4.3) | 29.1 (4.8) | <0.001 |
| **Waist circumference, cm** | 85.2 (12.9) | 85.0 (12.8) | 88.8 (12.7) | <0.001 |
| **Obesity based on BMI, n (%)** | 7,501 (20.4) | 6,529 (19.4) | 973 (36.3) | <0.001 |
| **Obesity based on WC, n (%)** | 7,665 (20.8) | 6,745 (19.8) | 920 (34.3) | <0.001 |
| **Obesity based on EITHER BMI or WC, n (%)** | 9,728 (26.4) | 8,564 (25.1) | 1,164 (43.4) | <0.001 |
| **Obesity status** |  |  |  | <0.001 |
| **No obesity based on both BMI and WC** | 27,056 (73.6) | 25,537 (74.9) | 1,519 (56.6) |  |
| **Obesity based on BOTH BMI and WC** | 5,438 (14.8) | 4,709 (13.8) | 729 (27.2) |  |
| **Misclassified as non-obese if obesity was defined by either BMI or WC alone** | 4,290 (11.7) | 3,855 (11.3) | 435 (16.2) |  |

UK, United Kingdom; BMI, body mass index; WC, waist circumference; Obesity based on BMI: defined as BMI ≥30 kg/m^2^; Obesity based on WC: defined as WC ≥88cm for men & ≥102cm for women

**Study limitation:
• Please note that the interpretation of PAF need to consider its well-known assumptions i.e. 1) the estimated ORs should be causal (which is not supported in observational studies), 2) the distribution of the other confounders regardless of obesity must be stable.**

Response: We agree with the reviewer that the assumption for PAF is that there is a causal relationship between the risk factors and the outcome. This is the assumption inherent whenever a PAF is calculated(9). PAF is most commonly calculated from data derived from observational studies despite this limitation. In our study, age, sex, country of birth, education all remained stable. Smoking status might change over time. However, this is a group of community-based population, and thus without any specific indication changes in smoking habit is low. Previous studies have suggested a relatively strong concordance of weight(10) and physical activity(11) over 10-20 years especially in a stable older population.

Author action: We have included the following statements in the limitations section (page 12, paragraph 1)

*The assumption for PAF calculation is that there is a causal relationship between the risk factor and outcome. PAF can be calculated using data from observational studies as long as this assumption is considered*(*22*)*. A further assumption in PAF is that the distribution of the other confounders regardless of obesity remains stable. Our key confounders of age, sex, and country of birth are stable. Studies have shown that there is a relatively strong concordance of levels of obesity and physical activity status over 20 years*(*23, 24*). *However, some misclassification may have occurred.*

**Reviewer #3: The paper is well writen and represents useful information for the clinician. BMI is frequently used for obesity screening. However, waist circumference seems more adequate in the evaluation of fat distribution. This paper indicates that, regarding knee OA, both measures indicate patients with higher risk of TKA for osteoarthritis. So, both can be used in clinical assessment.**

Response: We would like to thank the reviewer for these comments

**Reviewer #4: General comments:
The idea about “ Obesity defined by body mass index and waist circumference and risk of total knee arthroplasty for osteoarthritis” is interesting and original. Obesity is an important modifiable and on the other hand knee osteoarthritis as a common and significant orthopedic problem with subsequent knee arthroplasty. As a whole this manuscript is almost well written. However, I have some comments below:**

Response: We would like to thank the reviewer for these encouraging comments.

**Abstract:
The statement objective is not written well, it’s better to be written as that was at the end of the introduction.**

Author action: We have amended the aims in the abstract (page 2, paragraph 1).

*To examine the risk of total knee arthroplasty (TKA) due to osteoarthritis associated with obesity defined by body mass index (BMI) or waist circumference (WC) and whether there is discordance between these measures in assessing this risk.*

**Methods:
- The study design is a good and strength point and support the results of the study in addition to the large sample size.**

Response: Thank you for the encouraging words.

**- The subtitles of the methods is not suitable for this section, “Incidence of total knee arthroplasty for OA” and “Demographic data, anthropometric measurements and classification of obesity status” could be written in results section and not as a methodology subtitle. The subtitles in this section are study population with inclusion and exclusion criteria, Data collection as sampling, data collection tools,….etc**

Response: We have changed the subtitles as suggested by the reviewer.

Author action: We have changed the subtitles of the method section as suggested by the reviewer. Our current subtitles for the methods section are-

*Study population with inclusion and exclusion criteria*

*Data collection on total knee arthroplasty for OA*

*Data collection on demography and anthropometry*

**- Some details about the assessment of physical activity, questionnaires used are needed**

Response: Physical activity was assessed by three separate questions obtained from the Risk Factor Prevalence Study conducted by the National Heart Foundation and Australian Institute of Health regarding frequency of non-occupational vigorous and moderate physical activity, and walking(2, 4). Participants were categorised as whether or not participating in vigorous physical activity in line with values published in the Compendium of Physical Activities(5).

Author action: We have now clarified the issue raised by the reviewer and have amended the text in the manuscript. (page 5, paragraph 1)

*Physical activity was assessed by three separate questions obtained from the Risk Factor Prevalence Study conducted by the National Heart Foundation and Australian Institute of Health regarding frequency of non-occupational vigorous and moderate physical activity, and walking(12, 14). Participants were categorised as whether or not participating in vigorous activity in line with values published in the Compendium of Physical Activities(15).*

**- The absence of the flow chart of this cohort study is a great missing and should be included.**

Response: As we have included ~90% of the original participants in the statistical analysis and we have few missing data, we did not include a flow chart. As the reviewer has mentioned, the flow chart is given below.

41,514 participants

Recruited during 1990-94

Excluded (11.3%)

Left Australia or died before starting of follow-up (n=2599)

Reported having an arthroplasty prior to starting of follow-up or had a hip replacement n=2,094

Missing data on BMI or WC (n=37)

Included in the current analysis

n=36783

**- Inclusion of other factors as risk factors as secondary outcomes as: sex, age, physical activity and residence could be beneficial in this large study with large data.**

Response: We have included all these variables as confounders in the statistical analysis. Our aim was to examine the risk of severe knee OA assessed by total knee arthroplasty (TKA) due to OA associated with obesity defined by body mass index (BMI) or waist circumference (WC) and whether there is discordance between these measures in assessing this risk. We have examined the associations between exposure (obesity) and outcome (knee arthroplasty for OA) based on two regression models. Model 1 adjusted for age and sex, and Model 2 adjusted for age, sex, smoking status, vigorous physical activity and country of birth.

**Results:
- The titles of the tables and figures are short and are needed to be written in complete informative way especially of table (1).**

Response: As suggested, we have elaborated the title of table one. (page 7)

*Table 1. Demographic characteristics and obesity status of study participants at baseline (1990-94)*

**- In table (1):
• text comment there is a discrepancy between the total number of of participants with TKA and the number in table (1), please correct**.

Response: Thank you for pointing this out. This was an unintentional mistake. We have corrected this in the text.

**• It’s better to include female number with male number**

Response: As suggested, we have added the number of females in the table

|  | **Total population**  **(n=36,784)** | **No knee arthroplasty (n=34,101)** | **Knee arthroplasty**  **(n=2,683)** |
| --- | --- | --- | --- |
| **Age, years** | 62.6 (8.9) | 62.5 (8.9) | 63.7 (7.8) |
| **Male, n (%)** | 14,888 (40.5) | 13,976 (41.0) | 912 (34.0) |
| **Female, n (%)** | 21,896 (59.5) | 20,125 (59.0) | 1,771 (66.0) |

**• Clarify the numbers related to the BMI: AS if they are about mean+ SD, or what are they refer to? The same is in waist circumference.**

Response: As suggested by the reviewer, we have clarified this in the table.

This reads

| **BMI, kg/m^2^, mean (SD)** | 26.9 (4.4) | 26.7 (4.3) | 29.1 (4.8) |
| --- | --- | --- | --- |
| **Waist circumference, cm, mean (SD)** | 85.2 (12.9) | 85.0 (12.8) | 88.8 (12.7) |

**• Add the range (minimum – maximum) in age, BMI and waist circumference.**

Response: We checked the distribution of data on age, BMI and waist circumference. As these data were normally distributed, we presented mean and standard deviation. Several statistical publications suggested that, range is based on only two of the observations and may not be representative of the whole dataset, particularly if there are outliers which is very likely to be present in a large data set(12, 13). However, as the reviewer requested, we have presented these as an appendix.

Author action: The ranges of age, BMI and WC are presented in S2 Appendix*.*

S2 Appendix: The range (minimum – maximum) of age, body mass index and waist circumference

|  | **Total population**  **(n=36,784)** | **No knee arthroplasty (n=34,101)** | **Knee arthroplasty**  **(n=2,683)** |
| --- | --- | --- | --- |
| **Age, years** | 35.3 - 83.0 | 35.3 - 83.0 | 45.8 - 79.9 |
| **Body mass index, kg/m^2^** | 14.0 - 57.8 | 14.0 - 57.8 | 18.0 - 49.7 |
| **Waist circumference, cm** | 47.0 - 166.5 | 47.0 - 166.5 | 55.0 - 135.5 |

**Discussion
- After inclusion of other factors ( sex, age, physical activity and residence) could be included in the discussion section.**

Response: As suggested by the reviewer, we have added the following lines in the discussion section.

Author action: (page 10, paragraph 2)

*The results remained similar when we adjusted for age and sex (model 1) and further adjusted for* *smoking status, vigorous physical activity and country of birth (model 2).*

1.	Milne RL, Fletcher AS, MacInnis RJ, Hodge AM, Hopkins AH, Bassett JK, et al. Cohort Profile: The Melbourne Collaborative Cohort Study (Health 2020). Int J Epidemiol 2017;46:1757-i

2.	Jayasekara H, English DR, Haydon A, Hodge AM, Lynch BM, Rosty C, et al. Associations of alcohol intake, smoking, physical activity and obesity with survival following colorectal cancer diagnosis by stage, anatomic site and tumor molecular subtype. International journal of cancer 2018;142:238-50

3.	Siahpush M, English D, Powles J. The contribution of smoking to socioeconomic differentials in mortality: results from the Melbourne Collaborative Cohort Study, Australia. J Epidemiol Community Health 2006;60:1077-9

4.	MacInnis RJ, English DR, Hopper JL, Haydon AM, Gertig DM, Giles GG. Body size and composition and colon cancer risk in men. Cancer Epidemiol Biomarkers Prev 2004;13:553-9

5.	Ainsworth BE, Haskell WL, Leon AS, Jacobs DR, Jr., Montoye HJ, Sallis JF, et al. Compendium of physical activities: classification of energy costs of human physical activities. Medicine and science in sports and exercise 1993;25:71-80

6.	Shrier I, Platt RW. Reducing bias through directed acyclic graphs. BMC Med Res Methodol 2008;8:70

7.	Vandenbroucke JP, von Elm E, Altman DG, Gøtzsche PC, Mulrow CD, Pocock SJ, et al. Strengthening the Reporting of Observational Studies in Epidemiology (STROBE): explanation and elaboration. International journal of surgery (London, England) 2014;12:1500-24

8.	Vandenbroucke JP, von Elm E, Altman DG, Gøtzsche PC, Mulrow CD, Pocock SJ, et al. Strengthening the Reporting of Observational Studies in Epidemiology (STROBE): explanation and elaboration. Annals of internal medicine 2007;147:W163-94

9.	Mansournia MA, Altman DG. Population attributable fraction. BMJ (Clinical research ed) 2018;360:k757

10.	Hruby A, Hu FB. The Epidemiology of Obesity: A Big Picture. PharmacoEconomics 2015;33:673-89

11.	Guthold R, Stevens GA, Riley LM, Bull FC. Worldwide trends in insufficient physical activity from 2001 to 2016: a pooled analysis of 358 population-based surveys with 1·9 million participants. The Lancet Global health 2018;6:e1077-e86

12.	Whitley E, Ball J. Statistics review 1: presenting and summarising data. Critical care (London, England) 2002;6:66-71

13.	Soyemi K. Choosing the right statistical test. Pediatrics in review 2012;33:e38-44
